# Codelivery of Raloxifene and Rutin as PEGylated Nanoliposomes: Formulation, Characterization, and Prophylactic Activity Against Breast Cancer

**DOI:** 10.34172/apb.025.43681

**Published:** 2025-06-28

**Authors:** Maryam Abdulmaged Oleiwi, Ali Al-Samydai, Aya Y. Al-Kabariti, Khaldun M. Al Azzam, Simone Carradori, Walhan Alshaer

**Affiliations:** ^1^Pharmacological and Diagnostic Research Centre, Faculty of Pharmacy, Al-Ahliyya Amman University, Amman 19328, Jordan; ^2^Department of Biopharmaceutics and Clinical Pharmacy, Faculty of Pharmacy, Al-Ahliyya Amman University, Amman 19328, Jordan; ^3^Department of Chemistry, Faculty of Science, The University of Jordan, 11942, Amman, Jordan; ^4^Dipartimento di Farmacia, Università degli Studi Gabriele d’Annunzio Chieti-Pescara, Via dei Vestini 31, Chieti 66100, Italy.; ^5^Cell Therapy Center, The University of Jordan, 11942, Amman, Jordan

**Keywords:** Sustainability, Breast cancer, PEGylated, Nanoliposomes, Cytotoxicity, HPLC

## Abstract

**Purpose:**

Breast cancer is the leading cause of cancer-related deaths among women. Chemotherapy faces challenges such as systemic toxicity and multidrug resistance. Advances in nanotechnology have led researchers to develop safer and more efficient cancer treatment methods.

**Methods:**

The thin-film hydration method was employed to synthesize PEGylated nanoliposomes (NLs) loaded with raloxifene (RLX) and a combination of RLX and rutin. The NLs were characterized using a Zetasizer® instrument, transmission electron microscopy (TEM), and high-performance liquid chromatography (HPLC) analysis. The encapsulation of RLX and rutin was confirmed, and cell viability assays were conducted against breast cancer and normal endothelial cell lines.

**Results:**

The encapsulation efficiency significantly increased in the mixed formulation, with RLX reaching 91.28% and rutin 78.12%, indicating successful encapsulation. These NLs remained stable for up to two months at room temperature and one month at 4°C, demonstrating a biphasic release pattern. After 24 hours, approximately 17% of RLX was released from the NLs and 25% from the mixed NLs. In contrast, 55% of rutin was released from the NLs and 70.4% from the mixed NLs within 72 hours. The inclusion of rutin or RLX in the liposomal formulation reduced cytotoxicity against breast cancer cell lines, as indicated by the 3-(4,5-Dimethylthiazol-2-yl)-2,5-diphenyltetrazolium bromide (MTT) assay. However, it improved safety in normal human cells and tissues.

**Conclusion:**

PEGylated NLs loaded with RLX and rutin demonstrated safe anti-breast cancer effects, outperforming mixed NLs, suggesting the potential for a safer and more targeted treatment. Further investigations are needed into clinical translation.

## Introduction

 Cancer is characterized by atypical cell proliferation and dissemination, impacting life expectancy and healthcare systems.^1^ According to the World Health Organization, breast cancer (BC) is the most common and significant cause of cancer-related deaths. Treatment for BC depends on factors such as disease stage, receptor status, and patient preferences.^2^ It typically includes radiation therapy, chemotherapy, surgery, targeted therapy, immunotherapy, and hormonal therapy.^3^

 Ionizing radiation generates electrically charged particles, while chemotherapy targets cancer cells.^4^ Surgical methods include tumor removal, mastectomy, lymph node dissection, and axillary lymphatic system removal. Current treatment approaches emphasize tissue preservation and functional restoration through radiotherapy and imaging.^4,5^ Chemotherapy agents, such as anthracyclines and platinum-based drugs, induce cell death by disrupting DNA strands. While customized treatment plans are employed, side effects can be severe.⁶ The cyclophosphamide + methotrexate + 5-fluorouracil combination chemotherapy protocol helps reduce recurrence.

 Strategies for lowering BC risk include avoiding tobacco, minimizing hormone therapy and radiation exposure, and maintaining a healthy weight. Research has focused on personalized prevention strategies, precision medicine, immune system modulation, and the tumor microenvironment.⁷ Raloxifene (RLX), a selective estrogen receptor modulator, is used to prevent and treat osteoporosis in postmenopausal women.^8^

 It reduces the risk of invasive bone marrow cancer but may not decrease the risk of noninvasive bone cancer. RLX is contraindicated in patients with blood clots and it may increase the risk of deep vein thrombosis and pulmonary embolism. Common adverse effects include hot flashes and leg cramps.^9^

 Anticancer therapies often lack selectivity, leading to adverse effects such as anemia and neuropathy.^10^ Phytochemicals derived from plants exhibit anticancer properties and antioxidant activity. Flavonoids, glucosinolates, carotenoids, lignans, and resveratrol have been reported as potent antioxidants.^11^

**Figure 1:**
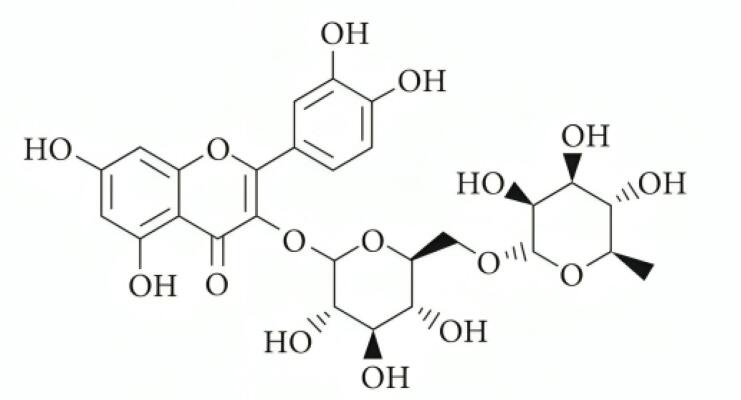
The chemical structure of rutin^21^

 Rutin (Figure 1), a bioactive compound found in citrus fruits, apples, berries, and tea leaves, was first identified in Ruta graveolens.^12,13^ Its pharmacological activities include managing Alzheimer’s disease, hyperkinetic movement disorders, and stroke, as well as preventing neuroinflammation and promoting neural crest cell survival.^14^ Rutin offers various health benefits, such as lowering hypertension, modulating blood coagulation, and preventing platelet aggregation.^15-18^ Additionally, it improves hair and skin health, acts as a natural sunscreen, supports atopic dermatitis management, enhances physical strength, and facilitates wound healing.^19,20^

 Rutin, a potent antioxidant, has potential as an anticancer drug due to its cytotoxic effects on cancer cells. These effects include inhibiting tumor growth, preventing proliferation, and inducing cell cycle arrest.^16^ The antiangiogenic properties of rutin limit tumor access to oxygen and nutrients.^17^ Additionally, rutin causes DNA damage in cancer cells, disrupting their genetic material and enhancing cytotoxicity. Its selective action minimizes potential side effects while increasing its effectiveness in cancer treatment. Combining rutin with conventional treatments such as chemotherapy or radiation therapy may enhance its cytotoxic effects. However, further clinical trials are needed to confirm its efficacy and safety.^18,19^

 Nanoliposomes (NLs) are small, spherical, or oval structures composed of a phospholipid bilayer, forming lipid vesicles ranging from 20 to 500 nanometers in size.^20^ Due to their biodegradability, non-toxicity, and non-immunogenic properties, biocompatible materials serve as efficient carriers for various drugs.^21,22^ Encapsulating drugs within NLs protects them from physiological degradation, enhancing their activity while reducing exposure to healthy tissue. The efficiency of NLs depends on their physicochemical properties, including size and charge. The use of synthetic phospholipids, such as 1,2-dioleoyl-sn-glycero-3-phospho-L-serine, has been employed to improve liposomal activity by modulating liposome structure and surface properties, generating negatively charged NLs.^23^

 Polyethylene glycol (PEG) is a highly hydrophilic and biocompatible polymer known for its excellent solubility in aqueous solutions, biocompatibility, and well-tolerated nature. The U.S. Food and Drug Administration (FDA) has approved PEG-conjugated pharmaceuticals for human use.^24^ PEGylation enhances material solubility but requires optimization for prolonged circulation. PEGs with molecular weights below 60 kDa tend to accumulate in the liver and lysosomes.^25^ The preparation of PEGylated NLs co-loaded with RLX and rutin may enhance selectivity, anticancer activity, and stability.

 Rutin exhibits antiplatelet activity,^26^ whereas RLX’s primary adverse effect is an increased risk of blood clot formation in the legs or lungs.^27^ Despite this risk, RLX is prescribed because its benefits are considered to outweigh its potential drawbacks, particularly for postmenopausal women at heightened risk of developing BC.^28^

 Combination therapy, which integrates pharmaceuticals with dietary supplements and natural compounds, may yield comparable outcomes to conventional chemotherapy but with fewer side effects.^29^ Traditional herbal therapies have demonstrated efficacy in treating nasopharyngeal, breast, and pancreatic cancers.^30^ Designing effective combination regimens requires a thorough understanding of cancer biology and potential drug interactions. Research and clinical studies indicate that combination therapy can improve cancer treatment outcomes and survival rates.^31-33^

 RLX, a selective estrogen receptor modulator, exhibits significant anticancer activity by binding to estrogen receptors in mammary tissue, thereby inhibiting DNA transcription. It functions as a chemopreventive agent, exerting estrogenic effects on bone, the cardiovascular system, breast tissue, and endometrium. RLX suppresses hormone-dependent BC cell proliferation, leading to apoptosis and cell cycle arrest. Postmenopausal women at elevated risk of BC may benefit from a five-year regimen of 60 mg/day.^34^ In mouse models of triple-negative breast cancer (TNBC), a daily oral dose of RLX inhibited tumor growth, promoted regression, reduced epidermal growth factor receptor (EGFR) expression, and diminished tumorigenicity in human TNBC cells.^35^ Furthermore, the combination of RLX and naringin increased antioxidant activity, suggesting that co-delivery via nanostructured lipid carriers could enhance therapeutic effectiveness and reduce side effects.^35^

 Molecular encapsulation within NLs is crucial for improving the stability and activity of pharmaceutical compounds. This method encapsulates active molecules within lipid bilayers, shielding them from enzymatic degradation and harsh environmental conditions.^36^ Park H proposed incorporating doxorubicin into NLs, evaluating its efficacy using two distinct formulations: Caelyx (pegylated liposomal doxorubicin hydrochloride) and Myocet (non-pegylated liposomal doxorubicin). These formulations exhibited comparable anticancer efficacy with reduced cardiotoxicity.^37^ Additionally, NLs significantly enhanced the antiproliferative effects of LPSF by encapsulating inclusion complexes, thereby increasing drug cytotoxicity.^38-40^

 This study aimed to investigate the anticancer and antioxidant properties of RLX and RLX-RUTIN-loaded NLs against MCF-7, MDA-MB-231, and EA. hy926 cells, with a focus on their selectivity. Furthermore, we explored the effects of NLs on co-delivering RLX and rutin. This research also developed a low-toxicity, BC-targeting NL formulation of RLX loaded into PEGylated liposomes. Additionally, the impact of rutin on drug loading, liposome size, and stability was examined.

## Materials and Methods

###  Materials

 RLX was obtained from Carbosynth UK/International (New Delhi, India). Rutin was purchased from Sygnus Biotech (Tokyo, Japan). Hydrogenated soybean phosphatidylcholine (HSPC) lipids, DSPE-PEG (2000) amine, and cholesterol were purchased from Avanti Polar Lipids (Alabama, USA). HPLC-grade chloroform and methanol were purchased from Across Organics (New Jersey, USA). Dulbecco’s phosphate-buffered saline (PBS) and Dulbecco’s modified eagle medium (DMEM) were obtained from Euroclone SpA (Figino, Italy). PBS tablets and concentrated phosphoric acid (85% w/w) were purchased from Fisher BioReagents (Pennsylvania, USA) and Sigma-Aldrich (Saint Louis, USA), respectively. Dimethyl sulfoxide (DMSO) and 70% alcohol were obtained from Fisher Chemical (Waltham, USA).

 The bromide (MTT) dye, Invitrogen 3-(4,5-dimethylthiazol-2-yl)-2,5-diphenyltetrazolium, was purchased from Thermo Fisher Scientific (Waltham, USA). 2,2-Diphenyl-1-picrylhydrazyl (DPPH) was obtained from SRLchem (Maharashtra, India). Roswell Park Memorial Institute (RPMI) medium was purchased from Euroclone SpA (Figino, Italy).

###  Instrumentation

 A digital balance (Ohaus Scales Adventurer) was used for weighing (Parsippany, NJ 07054, USA). A digital pH meter was purchased from Jenway (London, UK), while a centrifuge, microcentrifuge, CO₂ incubator, stirrer, sonicator, and water bath were acquired from Thermo Scientific (Waltham, MA, USA). The Buchi Rotavapor R-300 and a freeze dryer were utilized throughout the study (Flawil, Switzerland). A UV–visible spectrophotometer (UV-1800) was obtained from Shimadzu (Kyoto, Japan). A vortex mixer and mini extruder were purchased from VELP Scientifica (Velate MB, Italy), while a microscope was obtained from Nikon (Tokyo, Japan). A Nano Zetasizer was purchased from Malvern (Cambridge, UK). The probe sonicator was acquired from BANDELIN (Berlin, Germany). An ELISA microplate reader was obtained from BioTek (Santa Clara, USA). A Shimadzu HPLC system (Prominence-i LC-2030C Plus, Kyoto, Japan) was used for analysis. The HPLC unit was equipped with a UV-VIS Plus detector, a DGU-20A degasser, a SIL-20A autosampler, and a solvent delivery system pump. The Chrom Quest software (version 4.2.34) was used to record signals on an LC-Solution workstation (version 1.25, 2009–2010) (Shimadzu, Japan), running on Microsoft Windows XP.

###  Rutin determination using RP-HPLC

####  Chromatographic conditions

 The mobile phase consisted of a mixture of methanol and water (1:1, v/v), adjusted to pH 2.8 with concentrated phosphoric acid (85% w/w). The flow rate was set at 1 mL/min, and the instrument operated in isocratic mode. The mobile phase was prepared daily, degassed in a bath sonicator for 10 minutes, and filtered through a 0.45 μm filter paper before use. The column oven temperature was maintained at 40 °C, and separation was performed on a Fortis C18 column (150 mm × 4.6 mm, 5 µm) with UV detection at 287 nm and 360 nm for RLX and rutin, respectively. The injection volume was 10 µL.

####  Preparation of stock solution 

 Approximately 2 mg of rutin was weighed and dissolved in 2 mL of methanol to obtain a 1 mg/mL solution. The mixture was thoroughly vortexed, sonicated for 5 minutes, and then filtered through a 0.45 μm filter into HPLC vials for analysis using HPLC.^41^

####  Standard solutions for calibration curves 

 To prepare the stock solution, 10 mg of rutin was dissolved in 10 mL of methanol. A series of dilutions was then prepared by taking 5.0 mL of the stock solution and diluting it with 5.0 mL of methanol, yielding a total volume of 10 mL. This resulted in standard solutions with rutin concentrations of 1000, 500, 250, 125, 62.5, 31.25, and 15.625 µg/mL.

####  Determination of RLX using the RP-HPLC method

 The same chromatographic conditions were indicated above (chromatographic conditions).

####  Preparation of stock solution

 Two milligrams of RLX were weighed and dissolved in 2 mL of methanol (1 mg/mL), thoroughly mixed using vortexing, and filtered through a 0.45 μm filter.

####  Standard solutions for calibration curves

 After weighing 10 mg of RLX and dissolving it in 10 mL of methanol, a final stock solution with a concentration of 1 mg/mL was obtained. Serial dilutions were then prepared at concentrations of 1000, 500, 250, 125, 62.5, 31.25, and 15.625 µg/mL. The solutions were mixed thoroughly, filtered through a 0.45 μm filter, and analyzed to generate a calibration curve using Microsoft® Excel® workbook software. A linear formula was derived, and the coefficient of determination (R^2^) was calculated and used as a linearity parameter by ICH guidelines.

###  Preparation of PEGylated NLs using the thin film hydration method

 In a round-bottom flask, lipids along with rutin and/or RLX were accurately measured and dissolved in 5 mL of chloroform. To evaluate the impact of solvent variation, the results obtained using chloroform alone were compared with those from a chloroform-methanol mixture in a 4:1 % w/w ratio.^42-45^ All four NLs were prepared using the thin-film hydration method described by Al-Samydai et al.^46^ The specific quantities of lipids used for NL preparation are listed in Table 1.

**Table 1:** NLs formulations were prepared using the thin film method.

**Materials**	**Free**	**Formula 1**	**Formula 2**	**Formula 3**
HSPC (wt %)	55	55	55	55
DSPE/PEG 2000 (wt %)	5	5	5	5
Cholesterol (wt %)	40	40	40	40
RLX	-	20 mg	-	20 mg
Rutin	-	-	20 mg	20 mg

 The mixture was placed in a rotary evaporator at 50 °C with an initial pressure of 350 mbar, which was gradually reduced every 10 minutes until it reached 200 mbar. The process continued for 1 hour at a rotation speed of 70 rpm. Afterward, the mixture was allowed to evaporate, forming a thin film, and was then transferred to a -20 °C freezer for use the following day.

 The next day, the dried mixture was combined with a PBS solution by vortexing for 30 minutes, followed by continuous heating in a hot water bath. This ensured uniform suspension of all lipid components in the solution. The suspension was then incubated at 4 °C overnight to facilitate optimal lipid hydration.^45,47^

 Subsequently, the NLs were extruded using a mini extruder. The extrusion process was repeated 13 times to ensure the NLs exhibited a low polydispersity index (PDI). Unencapsulated compounds were removed by centrifugation at 7000 rpm, and the supernatant was collected for further analysis following the protocol described by Al-Samydai et al.^46^

###  Encapsulation efficiency and drug loading

 The degradation of NLs was carried out by adding 800 µL of methanol to 200 µL of the NLs, followed by bath sonication at 35 °C for 10 minutes. The mixture was then centrifuged at 12,000 rpm for 10 minutes. The supernatant was collected, filtered through a 0.45 μm syringe filter, and analyzed using HPLC.^46^


$$Encapsulation\;Efficiency(EE\%)=\frac{Entraped\;drug}{Total\;drug}\times 100\%$$

The percentage of drug loading was calculated as follows:


$$Drug\;loading(DL\%)=\frac{Weight\;of\;loaded\;drug}{Weight\;of\;lipids}\times 100\%$$

###  Characterization of the loaded NLs

 The NLs were characterized using dynamic light scattering (DLS) to determine their average size, PDI, and zeta potential. For analysis, each 50 μL sample was diluted with 1 mL of deionized water. The same procedure was followed for zeta potential measurement using a zeta potential measuring cuvette. The zeta potential and particle size were analyzed using Zetasizer software provided by Malvern Instruments. All samples were tested in triplicate to ensure precision. To assess the thermal stability of the formulation, the prepared NLs were stored at room temperature and in a refrigerator at 4 °C for two months.

###  In vitro drug release test

 In vitro release testing was conducted using the NL formulation, pure rutin, and RLX solutions. The membrane was blocked in PBS for 24 hours to remove the preservative before use. One milliliter of RLX, rutin-mixed NLs, or a pure solution of rutin and RLX was placed into a dialysis tubing cellulose membrane. The membrane was washed with 10 mL of PBS (pH 7.4) at 37 ± 0.5 °C in an aqueous bath under shaking at 100 rpm. One hundred microliters of the release medium were removed at fixed intervals (0.5, 1, 2, 4, 6, 24, 48, and 72 hours), replaced with the same amount of prewarmed PBS, and then injected into the HPLC system to obtain the following equation:


$$Release(\%)=\frac{Amount\;of\;drug\;released\;at\;time\;x}{Total\;amount\;of\;added\;drug}\times 100\%$$

###  Lyophilization of liposomal formulations

 Following liposome extrusion, the samples were stored at -70 °C for 24 hours, freeze-dried for an additional 24 hours, and then refrigerated at 4 °C for one week. The NLs were subsequently reconstituted in deionized water, and their stability was assessed using a Zetasizer.

###  Morphological study

 The morphology and structural configuration of the mixed NLs were examined using transmission electron microscopy (TEM). TEM imaging was performed using the negative staining technique.^48^ Initially, 200-mesh Formvar copper grids from SPI Supplies (USA) were subjected to carbon coating via a low-vacuum Leica EM ACE200 glow discharge coating machine (Leica, Austria). These carbon-coated grids were further treated with a 1.5% solution of Vinylec K in chloroform. A droplet of the liposome suspension, diluted with deionized water, was placed on the 200-mesh Formvar copper grid and allowed to air dry. The prepared grids were then stained with a 3% (v/v) aqueous solution of uranyl acetate for 20 minutes at an ambient temperature. After incubation, the grids were rinsed with distilled water, air-dried, and subsequently imaged using a Versa 3D TEM (FEI, Netherlands) operated at 30 kV.^46^

###  Cell viability assay (MTT)

 Two BC cell lines, estrogen receptor-positive (ER⁺) MCF-7 and estrogen receptor-negative (ER⁻) MDA-MB-231, along with the normal endothelial cell line EA. hy926, were seeded into 96-well plates (1 × 10⁴ cells/well) and incubated at 37 °C with 5% CO₂ for 24 hours. Rutin, RLX, and a combination of free and nanoliposomal formulations were applied in serial dilutions to determine the half-maximal inhibitory concentration (IC₅₀) using the MTT (3-(4,5-dimethylthiazol-2-yl)-2,5-diphenyltetrazolium bromide) assay.^49^

 The MTT assay measured the reduction of tetrazolium salt by mitochondrial dehydrogenases, producing a yellow tetrazolium compound proportional to the number of metabolically active viable cells. After 72 hours of drug exposure, MTT was added, and mitochondrial activity was evaluated after four hours. To assess the potential enhancement of RLX cytotoxicity within the nanoliposome formulation, cell proliferation was analyzed in formulations containing free RLX, rutin, mixed NLs, and PEGylated NLs.

###  Migration assay

 The ER⁺ MCF-7 and ER⁻ MDA-MB-231 BC cell lines were plated in sterile 6-well culture plates at a density of 800,000 cells per well and incubated at 37 °C with 5% CO₂ for 24 hours. The following day, a vertical scratch was made at the center of each cell monolayer using a sterile 1,000 µL micropipette tip to simulate a wound for free drug and NL treatment. Each well was then rinsed twice with sterile PBS.

 After 24 hours, cells were treated with RLX, RLX-loaded liposomes (RLX Lipo), a physical mixture, or mixed liposomes at a concentration of 0.5 × IC₅₀ or the IC₅₀ of the unencapsulated drug, as determined by the MTT assay. Images of the wound areas were captured at different time points using a phase-contrast microscope (model P. MICRO-001, Nikon) with a 4 × magnification objective. The wound closure area was measured using Motic Images Plus version 2.0 software, with a reference closure distance of 2 µm. DMSO and untreated culture media served as negative controls. The wound closure rate was assessed on day 1 (before treatment) and day 4 (72 hours post-treatment).^50^

 The percentage of wound closure was calculated using the following formula:


$$Rate\;of\;wound\;closur(\%)=\frac{Area\;for\;day1-Area\;for\;day4}{Area\;for\;day1}\times 100\%$$

###  In vitro antioxidant activity

 A DPPH solution was prepared by dissolving 2,2-diphenyl-1-picrylhydrazyl in methanol to a final concentration of 0.2 mM for the 96-well DPPH assay. Sample solutions containing RLX, rutin, and a physical mixture were prepared at varying concentrations using methanol as the solvent. Stock solutions (2 mg/mL) were first prepared for each component, followed by serial dilutions to obtain seven different concentrations. Similarly, serial dilutions were performed for the nanoformulations. The DPPH solution was added to the wells, followed by the samples were added to their respective wells, including blanks (methanol only) and vitamin C as the positive control. The microplate was incubated in the dark at room temperature for 30 minutes. Absorbance was then measured at approximately 517 nm using a microplate reader. The percentage inhibition (I%) of the DPPH free radical was calculated using the following equation^51^:


$$Antioxidant\;activity(\%)=1-\frac{Absorbance\;of\;sample}{Absorbance\;of\;control\;}\times 100\%$$

All tests were conducted three times, and the IC_50_ values are reported as the means ± SDs of triplicate samples.

###  Statistical analysis

 The results are expressed as the mean ± standard deviation from a minimum of three separate trials. Significance was assessed using various statistical tests (including paired *t-tests*, one-way ANOVA, and multiple repeated measures ANOVA). A p value < 0.05 was considered to indicate a statistically significant difference. The analyses were conducted using SPSS software (Version 21, IBM Corp.), GraphPad Prism 6 (GraphPad Software Inc., USA), and Microsoft Office Excel (Microsoft, USA).

## Results and Discussion

 The EE% was calculated, to ensure that only the supernatant contained the drug, an indirect method was used by measuring the drug concentration in the supernatant. The solubility of the drug in methanol under experimental conditions was confirmed. A standard solution was prepared in HPLC-grade methanol at the expected concentration for analysis. The solution was visually inspected for any signs of precipitation or undissolved particles and was found to be completely clear, indicating full solubility.

 Subsequently, the solution was analyzed using high-performance liquid chromatography (HPLC). Chromatographic analysis revealed a distinct and sharp peak corresponding to the drug, confirming its complete dissolution in methanol without any solubility issues. This validation establishes methanol as a suitable solvent for ensuring complete drug dissolution in the degradation and analysis procedure.

###  HPLC analysis

####  Qualitative and quantitative analysis of RLX

 Various conditions were optimized during method development to determine the most appropriate parameters for RLX analysis. Several wavelengths were tested, and to achieve high sensitivity, chromatographic separation was performed using an HPLC instrument (Shimadzu, Japan) with UV detection at a wavelength of 287 nm. The optimal mobile phase composition was determined to be 40% PBS and 60% acetonitrile (ACN), delivered isocratically at a flow rate of 1 mL/min. A 10 µL injection volume was used to generate a sharp peak.^1^

###  Validation

####  System suitability parameters

 The stock solution was introduced into the chromatographic system, and the system suitability parameters are presented in Table 2.

**Table 2:** The system suitability forHPLC parameters.

**No.**	**Parameters**	**RLX**
1	Retention time (t_r_)	2.392
2	Theoretical plate (*N*)	2344
3	Area (AUC) 1 mg/mL	13218933
4	Slope	2E + 07
5	Intercept	66803
6	Asymmetry (A_s_)	1.34
7	LLOD	0.001 mg/mL
8	LLOQ	0.015 mg/mL

RLX: Raloxifene; LLOD: The lower limit of detectionl; LLOQ: The lower limit of quantification.

####  Specificity

**Figure 2:**
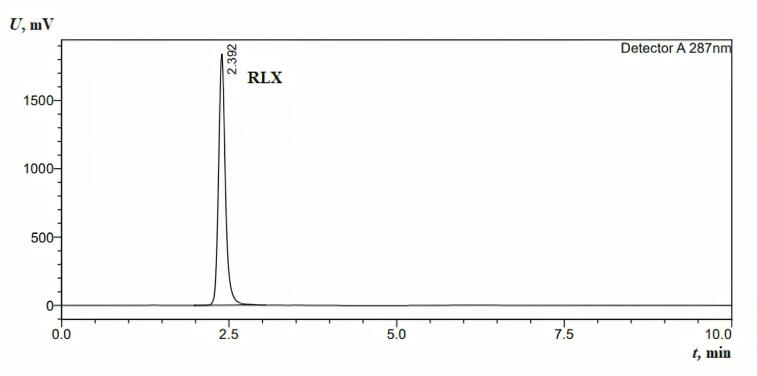
HPLC chromatogram for the standard injection of RLX (1 mg/mL). The mobile phase was as follows: methanol: water ratio of 1:1 (v/v), pH 2.8 (using concentrated phosphoric acid (85% w/w)); flow rate: 1 mL min^−1^ (isocratic mode); column oven: 40°C; column: Fortis C18 (150 mm × 4.6 mm, 5 µm); wavelength detection: 287 nm; and injection volume: 10 µL

 The method demonstrated specificity since there was no interference at the retention time corresponding to the analytical peak (Figure 2).

####  Linearity 

 The linearity range for the RLX calibration curves extended from 15.625 µg/mL to 1 mg/mL, with the curves plotted between peak area and concentration. The linear equation and correlation coefficient (R^2^) for RLX were y = 2E + 07x + 66803 and 0.9995, respectively. The resulting linear regression equation demonstrates a strong relationship between analyte concentration and peak area (response). The method’s sensitivity is represented by the slope (2E + 07), indicating a significant response to concentration variations. The correlation coefficient (R^2^ = 0.9995), being close to unity, signifies excellent linearity within the examined range. This strong R^2^ value confirms the calibration curve’s close fit to the experimental data with minimal deviation, reinforcing the accuracy and reliability of the analytical method for quantitative RLX determination.^52^

####  Precision

 In this method, the RSD was less than 2%, indicating that the method has good repeatability, with a mean of 1.85%. The low RSD value demonstrates that the method exhibits excellent repeatability, ensuring it can reliably produce comparable results across multiple trials. Precision is crucial for ensuring reliability in quantitative studies, particularly for methods intended for routine quality control, where reproducible results are essential for regulatory compliance and product safety.

###  RP-HPLC for rutin determination

**Figure 3:**
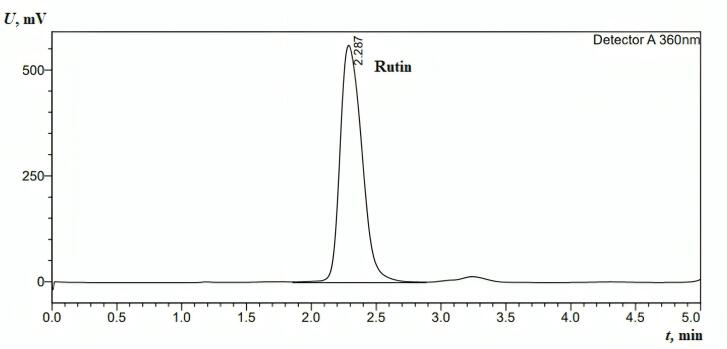
HPLC chromatogram for the standard injection of rutin (0.5 mg/mL).The mobile phase was as follows: methanol: water ratio of 1:1 (v/v), pH 2.8 (using concentrated phosphoric acid (85% w/w)); flow rate: 1 mL min^−1^ (isocratic mode); column oven: 40°C; column: Fortis C18 (150 mm × 4.6 mm, 5 µm); wavelength detection: 360 nm; injection volume:10 µL

 The linearity range for the rutin calibration curves, plotted between the peak area and concentration, was from 15.625 µg/mL to 1 mg/mL. The linear correlation coefficient for rutin was 0.9998. The absorbance was monitored at λ_max_ = 360 nm. The linear equation and correlation coefficient (R^2^) for rutin were y = 1E + 07x - 30539 and 0.9998, respectively. The method demonstrated specificity, as the retention time of the analytical peak remained unaffected by any interference (Figure 3).

###  Effect of solvent and rutin on encapsulation efficiency (EE%)

####  Evaluation of the effect of the solvent on the EE% of RLX

To assess the influence of the solvent used in the preparation process, adding methanol to the mixed formulation F3 resulted in a significant increase in the EE% of RLX, from 63.86 (standard deviation: 8.81) to 91.28 (standard deviation: 0.07). The data revealed unequal variances, as indicated by Levene’s test (*P* = 0.035). Further analysis of the different groups revealed significant differences, with a p-value of 0.033, as shown in Table 3.

**Table 3:** The effect of the solvent on the encapsulation efficiency (EE%) of RLX was assessed.

	**Formulation**	**Solvent**	**Mean**	**Standard deviation**	**Sig. (2-tailed)**
EE%	Mixed	With methanol	91.28	0.073	0.033
	Mixed	Without methanol	63.86	8.811	
	RLX	With methanol	51.97	2.410	0.114
	RLX	Without methanol	13.01	0.080	

RLX: Raloxifene; Mixed (Raloxifene and rutin).

There is a statistically significant difference (*P* < 0.05) between the mixed formulation with and without methanol, as indicated by the p-value of 0.033.

 However, although this difference is noteworthy, the p-value for RLX formulations is 0.114, indicating that this difference it is not statistically significant at the 0.05 level.

To assess the influence of the solvent used in the preparation process incorporating methanol into the formulation significantly improved in its EE, from 13.01 (standard deviation: 0.08) to 51.97 (standard deviation: 2.31). This substantial improvement highlights the critical role of solvent selection in optimizing the encapsulation process and enhancing drug loading efficiency. Methanol likely improved RLX solubilization and interactions with the encapsulating material, thereby increasing overall drug entrapment within the formulation matrix. The data indicated equal variances, as demonstrated by Levene’s test (*P* = 0.114). Further analysis of the different groups revealed significant differences, with a p-value of *P* ≤ 0.001, as shown in Table 3.

####  Effect of rutin on the encapsulation efficiency of RLX

 To assess the influence of rutin in the preparation process, its addition to the formulation of RLX led to a significant increase in the EE of RLX, from 51.97 (standard deviation: 2.3) to 91.28 (standard deviation: 0.07). This enhancement highlights rutin’s essential role in optimizing the encapsulation process. The substantial increase in EE suggests that rutin may contribute to stabilizing RLX within the encapsulating matrix, possibly due to its antioxidant properties or its influence on the structural integrity of the delivery system.

 The data indicated unequal variances, as evidenced by Levene’s test p-value of 0.032. Further analysis of the different groups revealed significant differences, with a *P* value of 0.001, as shown in Table 4, confirming rutin’s positive effect on EE.

**Table 4:** Effect of rutin on the encapsulation efficiency of RLX

	**Formulation**	**Mean**	**Standard deviation**	**Sig. (2-tailed)**
Encapsulation Efficiency (EE%)	With Rutin	91.29	0.073	0.001
	Without Rutin	51.98	2.317	

 The difference in EE between formulations with and without rutin is statistically significant, as indicated by the highly significant *P* value (0.001) (*P* < 0.05). This demonstrates that rutin is a crucial component of the formulation, exerting a noticeable and meaningful effect in enhancing EE%.

####  Effect of solvent on the EE% of rutin alone

 To assess the influence of methanol addition on the EE% of rutin alone, the incorporation of methanol into the formulation resulted in a significant increase in the EE% of rutin, rising from 67.84 (standard deviation: 0.045) to 78.12 (standard deviation: 0.39). The data indicated unequal variances, as evidenced by Levene’s test (*P* = 0.006). Further analysis of the different groups revealed significant differences, with a *P* value ≤ 0.001, as shown in Table 5. The observed difference is statistically significant and unlikely to be due to chance, as indicated by the highly significant *P* value ( ≤ 0.001). These findings demonstrate that methanol plays a crucial role in enhancing encapsulation efficiency.

**Table 5:** Impact of solvent on the encapsulation efficiency (EE%) of rutin alone.

	**Formulation**	**Mean**	**Standard deviation**	**Sig. (2-tailed)**
EE%	Without Methanol	67.84	0.045	≤ 0.001
	With Methanol	78.12	0.398	

####  Effect of solvent on the EE% of rutin in the mixed formulation

 To evaluate the impact of methanol on the EE% of rutin in the mixed formulation, its effect was analyzed during the preparation process. The addition of methanol led to a significant reduction in EE%, decreasing from 38.11% (SD: 0.12) to 21.03% (SD: 0.98). This decline suggests that methanol disrupts the encapsulation process under the given conditions. Its solvent properties may interfere with rutin’s interaction with the encapsulating material, potentially altering solubility, weakening hydrophobic interactions, or inducing premature drug leakage.

 Levene’s test confirmed unequal variances (*P*= 0.002), and further statistical analysis revealed a highly significant difference between the groups (*P* ≤ 0.001), as presented in Table 6. These findings indicate that methanol adversely affects rutin encapsulation efficiency in mixed formulations. The strong statistical significance (*P* = 0.001, *P* < 0.05) confirms a notable difference in EE% between the conditions, reinforcing methanol’s detrimental impact on encapsulation efficiency in this formulation.

**Table 6:** Effect of solvent on rutin’s encapsulation efficiency (EE%) in the mixed (raloxifene and rutin) formulation.

	**Formulation**	**Mean**	**Standard deviation**	**Sig. (2-tailed)**
EE%	Without Methanol	38.11	0.121	0.001
	With Methanol	21.03	0.987	

####  Effect of mixture formation on the EE% of rutin in the mixed formulation 

 To assess the impact of RLX on the EE% of rutin during the preparation process, RLX was incorporated into the formulation. This addition led to a significant reduction in EE%, decreasing from 78.12 (SD: 0.39789) to 38.11 (SD: 0.121). The substantial decline suggests that RLX interacts with the encapsulating matrix or competes with rutin for entrapment sites, thereby reducing the system’s capacity to retain both compounds effectively.

 Levene’s test indicated unequal variances (*P* = 0.015), and further analysis confirmed significant differences between groups, with a *P* value of ≤ 0.001 (Table 7). Since this *P* value is highly significant (*P* < 0.05), the observed reduction in EE% is unlikely due to chance. This finding highlights RLX as a key factor in lowering encapsulation efficiency within this formulation.

**Table 7:** Effect of the mixture on the encapsulation efficiency (EE%) of rutin in the mixed (raloxifene and rutin) formulation.

	**Formulation**	**Mean**	**Standard deviation**	**Sig. (2-tailed)**
EE%	Without RLX	78.12	0.398	*P* ≤ 0.001
	With RLX	38.11	0.121	

RLX: Raloxifene.

###  Nanoformulation characterization

####  Characterization of the particle size, PDI, and charge of NLs

 The average size, PDI, and charge of the freshly prepared NLs were assessed, with measurements taken in triplicate for each run.

 This study evaluated the impact of loading materials on nanoparticle characterization. The results showed that the particle size of mixed-loaded NLs (F3) was 125.38 nm, RLX-loaded NLs (F1) 138.25 nm, and free NLs 123.56 nm. ANOVA, followed by the least significant difference (LSD) test, revealed significant differences among the groups (*P* < 0.05), as shown in Table 8. However, no significant difference was observed between co-loaded NLs and free NLs (*P* = 0.567). In contrast, RLX-loaded NLs showed significantly larger particle sizes compared to both co-loaded and free NLs (*P* < 0.05). Notably, all formulations remained within the optimal size range for NLs ( < 300 nm), ensuring their suitability for drug delivery applications.

**Table 8:** Influence of loading on the characterization of NLs

**Parameter**	**NLs**	**Mean**	**Standard Deviation**	**ANOVA**		**Multiple Comparisons LSD**		
				**F**	**Sig.**			
Size (nm)	Co Loaded NLs	125.38	1.51	17.32	*P* ≤ 0.001	Co Loaded NLs	RLX Loaded Nanoliposomes	*P* ≤ 0.001
	RLX Loaded Nanoliposomes	138.25	6.51			-	Free Nanoliposomes	0.567
	Free Nanoliposomes	123.57	1.59			RLX Loaded Nanoliposomes	Free Nanoliposomes	*P* ≤ 0.001
PDI	Co Loaded NLs	0.124	0.020	12.76	0.002	Co Loaded NLs	RLX Loaded Nanoliposomes	0.205
	RLX Loaded Nanoliposomes	0.141	0.024	12.76	0.002	-	Free Nanoliposomes	0.001
	Free Nanoliposomes	0.193	0.006			RLX Loaded Nanoliposomes	Free Nanoliposomes	0.006
Zeta potential (mV)	Co Loaded NLs	-10.75	1.13	33.52	*P* ≤ 0.001	Co Loaded NLs	RLX Loaded Nanoliposomes	*P* ≤ 0.001
	RLX Loaded Nanoliposomes	-4.25	1.90	33.52	*P* ≤ 0.001	-	Free Nanoliposomes	*P* ≤ 0.001
	Free Nanoliposomes	-5.04	0.650	33.52	*P* ≤ 0.001	RLX Loaded Nanoliposomes	Free Nanoliposomes	0.454

PDI: The polydispersity index; RLX: Raloxifene; NLs: Nanoliposomes; LSD: Fisher's least significant difference; F: F-Statistic.

 The PDI values were 0.1237 for mixed-loaded NLs, 0.1408 for RLX-loaded NLs, and 0.1930 for free NLs. ANOVA and LSD testing indicated significant differences among the groups (*P* < 0.05). While mixed-loaded and RLX-loaded NLs showed no significant difference (*P* = 0.205), free NLs significantly differed from both (*P* < 0.05). Importantly, all formulations maintained a PDI below 0.300, confirming homogeneous size distribution and enhanced stability. The lower PDI of mixed-loaded NLs suggests improved uniformity when RLX and rutin are combined, as presented in Table 8.

 The zeta potential, a crucial indicator of surface charge and stability, measured -10.7 mV for mixed-loaded NLs, -4.2 mV for RLX-loaded NLs, and -5.04 mV for free NLs. ANOVA and LSD testing revealed significant differences among the groups (*P* < 0.05), except between free NLs and RLX-loaded NLs (*P* = 0.454). Co-loaded NLs exhibited a significantly greater negative charge than the other formulations (*P* < 0.05). All formulations-maintained zeta potential values within the optimal range (-20 to + 20 mV), ensuring sufficient electrostatic repulsion for colloidal stability. The higher negative charge of mixed-loaded NLs suggests improved stability, likely due to the combined effects of RLX and rutin on surface charge properties, as indicated in Table 8.

####  Examination of nanoliposome stability at 25 °C

 The stability of NLs was evaluated over two months under storage conditions at 25 °C, focusing on key parameters such as PDI, particle size (nm), and zeta potential (mV). Stability is a critical factor in determining the feasibility of NL formulations for long-term storage and pharmaceutical applications.

**Figure 4:**
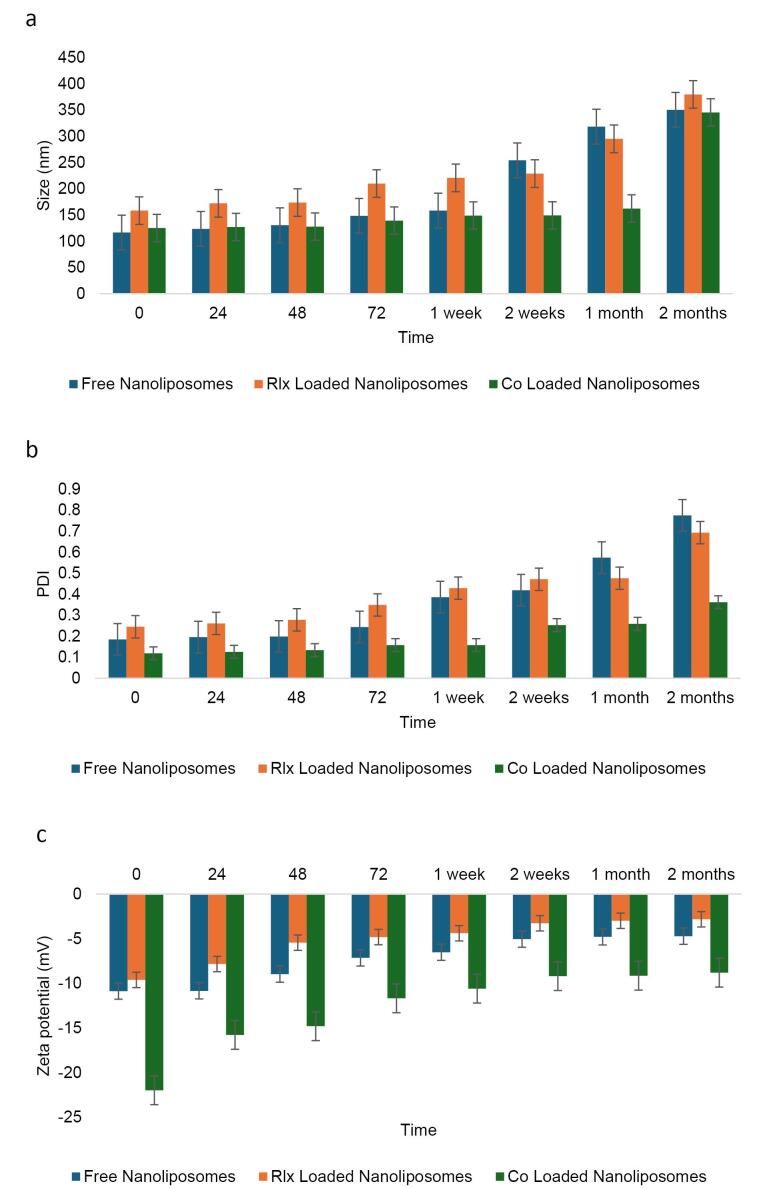
Stability of the NLsover two months under storage at 25°C. a:Size change, b: PDI change, and c: charge change

 Size analysis revealed that free NLs exhibited values beyond the acceptable range after just one week and continued to be unstable throughout the two month (Figure 4a). In contrast, RLX-loaded NLs remained stable for up to two months, while co-loaded NLs stayed within acceptable limits for the entire storage duration at 25 °C. Similarly, PDI measurements indicated that free NLs became unstable after one week, whereas co-loaded NLs showed instability within 72 hours. Meanwhile, RLX-loaded NLs maintained stability for up to one month (Figure 4b).

 Regarding zeta potential, all formulations—free NLs, RLX-loaded NLs, and co-loaded NLs—remained within the optimal range for NL formulations (Table 9). Throughout the storage period, zeta potential values, which indicate surface charge and colloidal stability, were maintained within the permissible range of -20 to + 20 mV (Figure 4c). The specific values suggest sufficient electrostatic repulsion to prevent significant aggregation: -11.45 mV for co-loaded NLs, -3.91 mV for RLX-loaded NLs, and -1.14 mV for free NLs. Despite having the lowest absolute zeta potential, free NLs exhibited instability in both size and PDI, suggesting that electrostatic stabilization alone was insufficient to maintain long-term stability. The absence of additional stabilizing interactions found in RLX-loaded or co-loaded systems likely contributed to this instability.

**Table 9:** Examination of nanoliposome stability at 25°C.

**Parameter**	**NLs**	**Mean**	**Standard deviation**	**ANOVA**		**Multiple Comparisons LSD (** ***P*** ** value)**		
				**F**	**Sig.**	**Test group**	**Compared group **	**Sig.**
Size (nm)	Co loaded NLs	144.94	10.54	20.75	*P* ≤ 0.001	Co loaded NLs	RLX Loaded Nanoliposomes	0.316
	RLX loaded nanoliposomes	160.44	18.57			-	Free Nanoliposomes	0.000
	Free nanoliposomes	237.07	83.27			RLX loaded nanoliposomes	Free Nanoliposomes	*P* ≤ 0.001
PDI	Co loaded NLs	0.249	0.074	11.94	*P* ≤ 0.001	Co loaded NLs	RLX Loaded Nanoliposomes	0.297
	RLX loaded nanoliposomes	0.298	0.095			-	Free Nanoliposomes	*P* ≤ 0.001
	Free nanoliposomes	0.467	0.234			RLX loaded nanoliposomes	Free Nanoliposomes	0.001
Charge (mV)	Co loaded NLs	-11.45	1.960	144.7	*P* ≤ 0.001	Co loaded NLs	RLX Loaded Nanoliposomes	*P* ≤ 0.001
	RLX loaded nanoliposomes	-3.910	1.400			-	Free Nanoliposomes	*P* ≤ 0.001
	Free nanoliposomes	-1.140	2.570			RLX loaded nanoliposomes	Free nanoliposomes	*P* ≤ 0.001

PDI: Polydispersity index; RLX: Raloxifene; NLs: Nanoliposomes; LSD: Fisher's least significant difference; F: F-Statistics

 Statistical analysis of NL characteristics, including size, PDI, and surface charge, revealed significant differences among formulations, as confirmed by ANOVA and LSD multiple comparisons. Particle size analysis (F = 20.75, *P* ≤ 0.001) showed that free NLs (237.07 nm, SD = 83.27) were significantly larger than both co-loaded NLs (144.94 nm, SD = 10.54) and RLX-loaded NLs (160.44 nm, SD = 18.57). However, the difference between co-loaded and RLX-loaded NLs was not statistically significant (*P* = 0.316), suggesting that RLX incorporation had minimal impact on particle size, whereas free NLs exhibited significantly greater size variability and heterogeneity (*P* ≤ 0.001).

 The last three columns show the significant pairwise comparisons obtained using the ANOVA multiple comparison test. Specifically, numbers are the p-value for the comparison of free NLs to the appropriate comparison group.

 PDI analysis (F = 11.94, *P* ≤ 0.001) further confirmed that free NLs had the highest polydispersity index (0.467, SD = 0.234), significantly differing from RLX-loaded NLs (0.298, SD = 0.095, *P* = 0.001) and co-loaded NLs (0.249, SD = 0.074, *P* ≤ 0.001). However, no significant difference was observed between RLX-loaded and co-loaded NLs (*P* = 0.297), indicating that both formulations maintained a relatively uniform size distribution. In contrast, free NLs exhibited the highest heterogeneity, which could lead to instability and aggregation.

 Zeta potential measurements (F = 144.7, *P* ≤ 0.001) indicated that co-loaded NLs (-11.45 mV, SD = 1.960) had the most stable surface charge, significantly differing from RLX-loaded NLs (-3.91 mV, SD = 1.400, *P* ≤ 0.001) and free NLs (-1.14 mV, SD = 2.570, *P* ≤ 0.001). The lower zeta potential of RLX-loaded and free NLs suggested weaker electrostatic repulsion, increasing the risk of aggregation. Additionally, the large variation in zeta potential within free NLs (SD = 2.570) further confirmed their poor stability.

####  Examination of NL stability at 4°C

**Figure 5:**
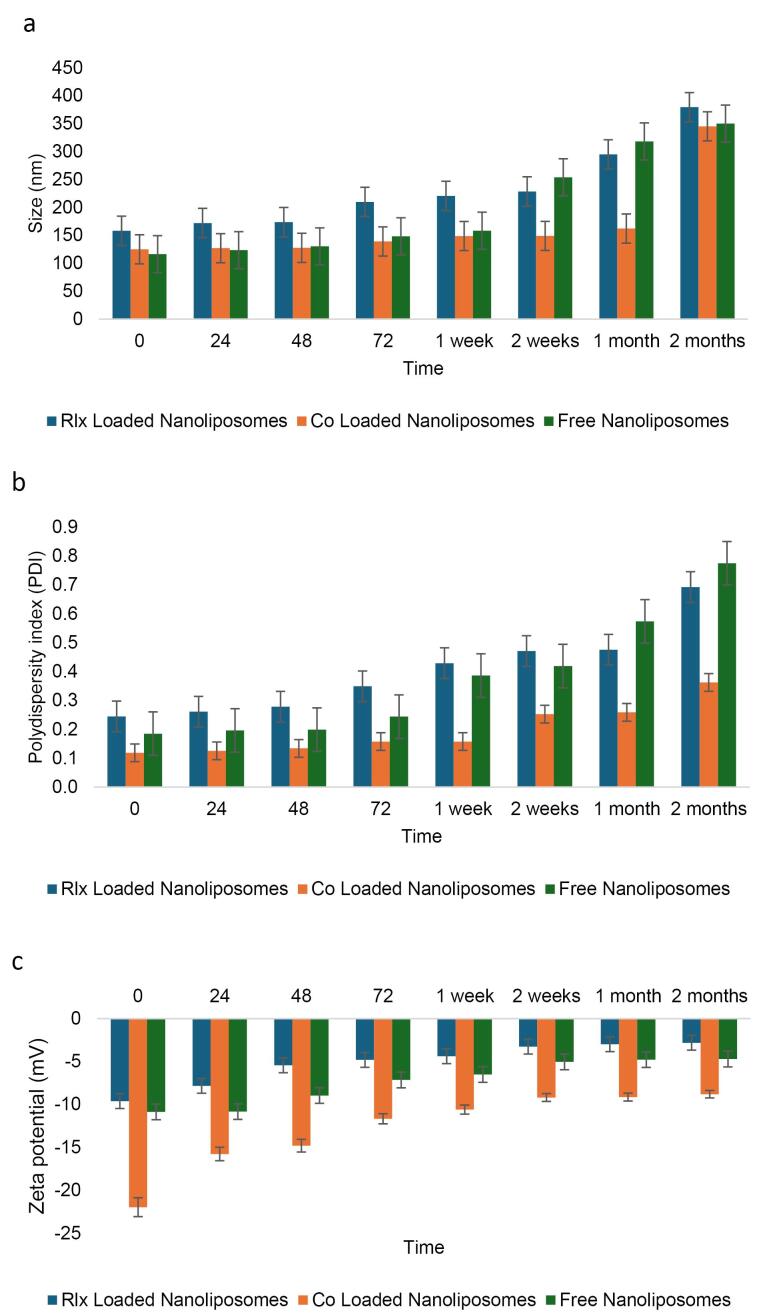
Stability of the NLsover two months under storage at 4°C. a:Size change, b: PDI change, and c: charge change

 The stability of NLs was assessed under storage conditions at 4°C for two months. Key parameters, including PDI, particle size (nm), and zeta potential (mV), were measured, yielding p-values of 0.307, 0.036, and 0.001, respectively. Maintaining stability at low temperatures is essential for preserving the efficacy of nanoparticle-based formulations. Size analysis indicated that both free NLs and RLX-loaded NLs remained stable for up to two weeks, while co-loaded NLs exhibited stability for up to one month. Despite some size increases over time due to potential aggregation or structural rearrangements, all formulations remained within acceptable limits at 4°C (Figure 5a). This suggests that the combination of RLX and rutin may enhance structural stability by modifying the rigidity and composition of the lipid bilayer in the co-loaded system.

 Similarly, free NLs exhibited instability in PDI values after just two weeks of storage, whereas the PDI of co-loaded NLs remained stable for up to two months (Figure 5b). In contrast, RLX-loaded NLs maintained stable PDI values for only one week (Figure 5c, Table 10). Regarding zeta potential, all formulations fell within the optimal range for nanoliposome stability. Notably, co-loaded NLs displayed a higher negative charge (-12.74 mV) compared to free NLs (-7.35 mV) and RLX-loaded NLs (-5.14 mV) (Figure 5c). Over time, the greater absolute zeta potential of co-loaded NLs likely contributed to enhanced electrostatic stabilization, minimizing particle aggregation and improving colloidal stability.

**Table 10:** Examination of nanoliposome stability at 4°C

**Parameter**	**NLs**	**Mean**	**Standard deviation**	**ANOVA**		**Multiple Comparisons LSD (** ***p*** ** value)**		
				**F**	**Sig.**	**Test group**	**Compared group**	**Sig.**
Size (nm)	RLX Loaded NLs	229.76	74.42	1.251	0.307	RLX Loaded NLs	Co Loaded NLs	0.129
	Co Loaded NLs	165.60	73.81			-	Free NLs	0.471
	Free NLs	199.99	93.78			Co Loaded NLs	Free NLs	0.407
PDI	RLX Loaded NLs	0.400	0.150	3.892	0.036	RLX Loaded NLs	Co Loaded NLs	0.018
	Co Loaded NLs	0.196	0.086			-	Free NLs	0.728
	Free NLs	0.372	0.213			Co Loaded NLs	Free NLs	0.038
Zeta potential (mV)	RLX Loaded NLs	-5.140	2.440	10.98	0.001	RLX Loaded NLs	Co Loaded NLs	*P* ≤ 0.001
	Co Loaded NLs	-12.74	4.560	-	-	-	Free NLs	0.199
	Free NLs	-7.350	2.580			Co Loaded NLs	Free NLs	0.004

PDI: The polydispersity index; RLX: Raloxifene; NLs: Nanoliposomes; LSD: Fisher's least significant difference.

 The ANOVA results (F = 1.251, *P* = 0.307) indicate no statistically significant difference in particle size among the three formulations. RLX-Loaded NLs (229.76 nm, SD = 74.42), Co-Loaded NLs (165.60 nm, SD = 73.81), and Free NLs (199.99 nm, SD = 93.78) exhibit considerable variation; however, multiple comparisons reveal that none of the pairwise differences are statistically significant (*P* > 0.05). This suggests that the incorporation of RLX or co-loading does not significantly impact overall particle size. The relatively high standard deviations indicate a broad distribution of particle sizes, which may influence formulation stability.

 The values in the last three columns represent the significant pairwise comparisons obtained from the ANOVA multiple comparison test. Specifically, the 0.471 value indicates the p-value for the comparison between the free NLs and the respective comparison group. The value 1.251 refers to the F-statistic obtained from the ANOVA test, which measures the ratio of variance between the groups. The value 0.307 is the corresponding p-value, which indicates the level of statistical significance. Since the p-value is greater than the typical threshold (e.g., *P* ≤ 0.05), it suggests that the differences between the groups are not statistically significant.

 The PDI measures the uniformity of particle size distribution. ANOVA results (F = 3.892, *P* = 0.036) indicate a statistically significant difference among the formulations. Co-loaded NLs (0.196, SD = 0.086) exhibit the lowest PDI, suggesting a more uniform and stable formulation. In contrast, RLX-loaded NLs (0.400, SD = 0.150) and free NLs (0.372, SD = 0.213) have significantly higher PDI values, reflecting greater size heterogeneity. Multiple comparisons confirm significant differences between RLX-loaded NLs and co-loaded NLs (*P* = 0.018) and between co-loaded NLs and free NLs (*P* = 0.038). However, no significant difference is observed between RLX-loaded NLs and free NLs (*P* = 0.728). These findings suggest that co-loading enhances particle uniformity, whereas RLX-loaded and free formulations exhibit greater heterogeneity.

 Zeta potential is a key indicator of colloidal stability. ANOVA results (F = 10.98, *P* = 0.001) reveal a highly significant difference among the formulations. Co-loaded NLs (-12.74 mV, SD = 4.560) exhibit the most negative charge, indicating stronger electrostatic repulsion and greater colloidal stability. In contrast, RLX-Loaded NLs (-5.140 mV, SD = 2.440) and Free NLs (-7.350 mV, SD = 2.580) display significantly lower negative charges, suggesting weaker repulsive forces and a higher tendency for aggregation. Multiple comparisons confirm highly significant differences between RLX-loaded NLs and Co-loaded NLs (*P* ≤ 0.001) and between Co-Loaded NLs and Free NLs (*P* = 0.004). However, no significant difference is observed between RLX-loaded NLs and free NLs (*P* = 0.199), indicating that both formulations exhibit similar colloidal stability, which is lower than that of co-loaded NLs.

####  Lyophilization stability 

 A paired t-test was conducted to assess the impact of lyophilization on the characterization parameters of the co-loaded NLs, including size (nm), PDI, and zeta potential (mV) (Table 11). Significant differences were observed, with p-values of 0.023, 0.001, and 0.03 for size, PDI, and zeta potential, respectively. These findings indicate that the structural properties of the NLs were notably affected by the freeze-drying process. However, the size and charge of the NLs remained within the optimal range after lyophilization.

**Table 11:** The influence of lyophilization on the characterization parameters includes size (nm) change, PDIchange, and charge change

**Condition**	**Particle size (nm)**	**PDI **	**Zeta potential (mV)**
Before lyophilization	250	0.21	-6
After lyophilization	320	0.45	-11

PDI: Polydispersity Index.

 Despite these changes, both the size and zeta potential remained within acceptable limits for nanoparticle stability post-lyophilization. Maintaining a particle size below 300 nm is crucial for enhancing drug bioavailability and cellular uptake, while a zeta potential within the range of -20 mV to + 20 mV provides sufficient electrostatic repulsion to prevent aggregation. The ability of the NLs to retain these desirable properties suggests that their overall colloidal stability was not significantly compromised, supporting the lyophilized formulation’s potential for long-term storage and transport. However, the PDI exceeded 0.3, possibly due to the absence of sucrose in the lyophilization process.

###  In vitro drug release assay

**Figure 6:**
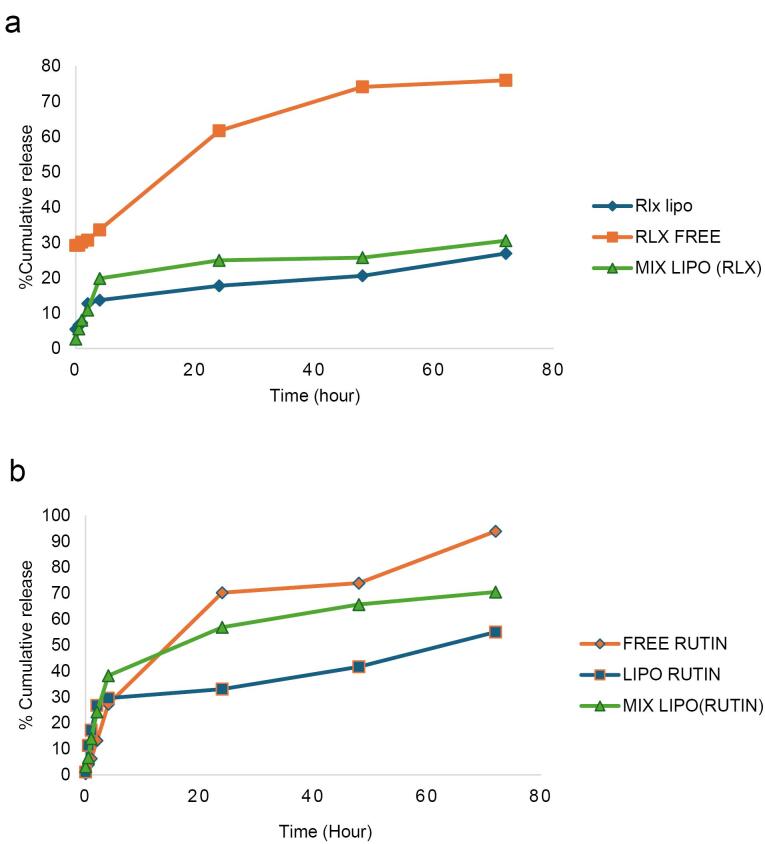
In vitro release assay of a: RLX and b: rutin over 70 hours

 The release rate of RLX from NLs was significantly slower than that from the free RLX solution (Figure 6a). The results indicate that RLX NLs exhibited a distinctive biphasic release profile, characterized by an initial burst phase followed by a considerably slower release phase. During the first two hours, RLX molecules located on the lipid bilayer surface and not fully encapsulated within the NLs contributed to the initial burst release. After four hours, the amount of RLX released from the free solution reached approximately 33.6% ± 1.61. However, after 24 hours, only 17% ± 0.97 of RLX was released from RLX NLs, compared to around 25% ± 2.21 from mixed NLs. In comparison, 93.8% ± 1.07 of rutin was liberated in the free solution within 72 hours, whereas 55% ± 1.98 was released from the rutin NLs and 70.4% ± 1.20 from the mixed NLs (Figure 6b). The increased release from mixed NLs suggests that RLX-rutin interactions may influence the structural permeability of the liposomal membrane. The continuous release profile observed in NLs aligns with previously published findings.^53,54^ The sustained release characteristics of NLs can be attributed to the integration of RLX and rutin within the lipid bilayer, which restricts their rapid diffusion into the dialysate. Moreover, the encapsulation approach offers multiple advantages, including enhanced bioavailability, reduced dosing frequency, and minimized dose-dependent RLX toxicity. Consequently, encapsulating RLX and rutin in NLs may serve as an effective strategy for the sustained delivery of rutin in the body while simultaneously mitigating RLX’s dose-dependent toxicity.^55^

###  Morphological study

####  Transmission electron microscopy (TEM) 

**Figure 7:**
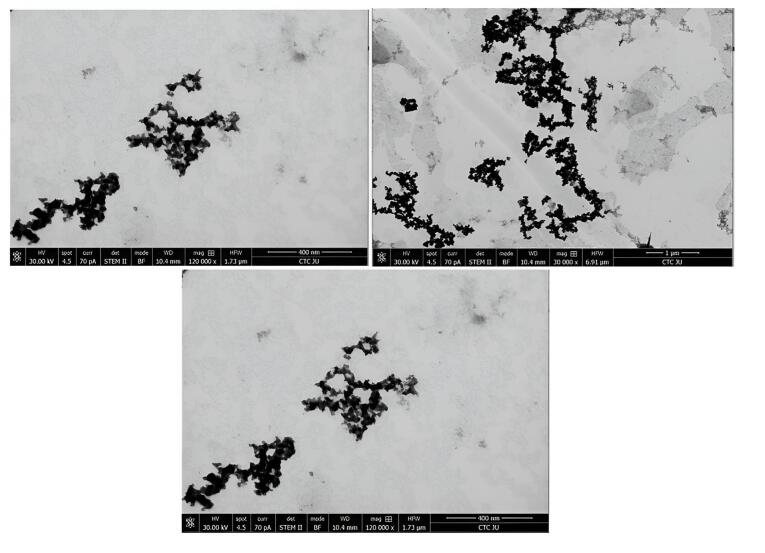
TEM shape and size of the mixed NLs

 TEM provided valuable insights into the morphology and size distribution of the mixed NLs. TEM analysis revealed that the mixed NLs exhibited a uniform structure with a smooth, spherical shape and an average size of 100 ± 30.4 nm (n = 15). As shown in Figure 7, RLX and rutin were successfully encapsulated within the NLs. The TEM images clearly demonstrated the incorporation of RLX and rutin into the nanoliposome structure, confirming their successful entrapment. The localization of these active compounds depends on their solubility properties: RLX, being relatively hydrophobic, is likely associated with the lipid bilayer, whereas rutin, possessing both hydrophobic and hydrophilic regions, may be distributed between the lipid bilayer and the aqueous core. This co-loading strategy enhances the potential for synergistic therapeutic effects and controlled drug release.

###  Cell viability assay 

####  ER-positive BC cell line (MCF-7)

**Figure 8:**
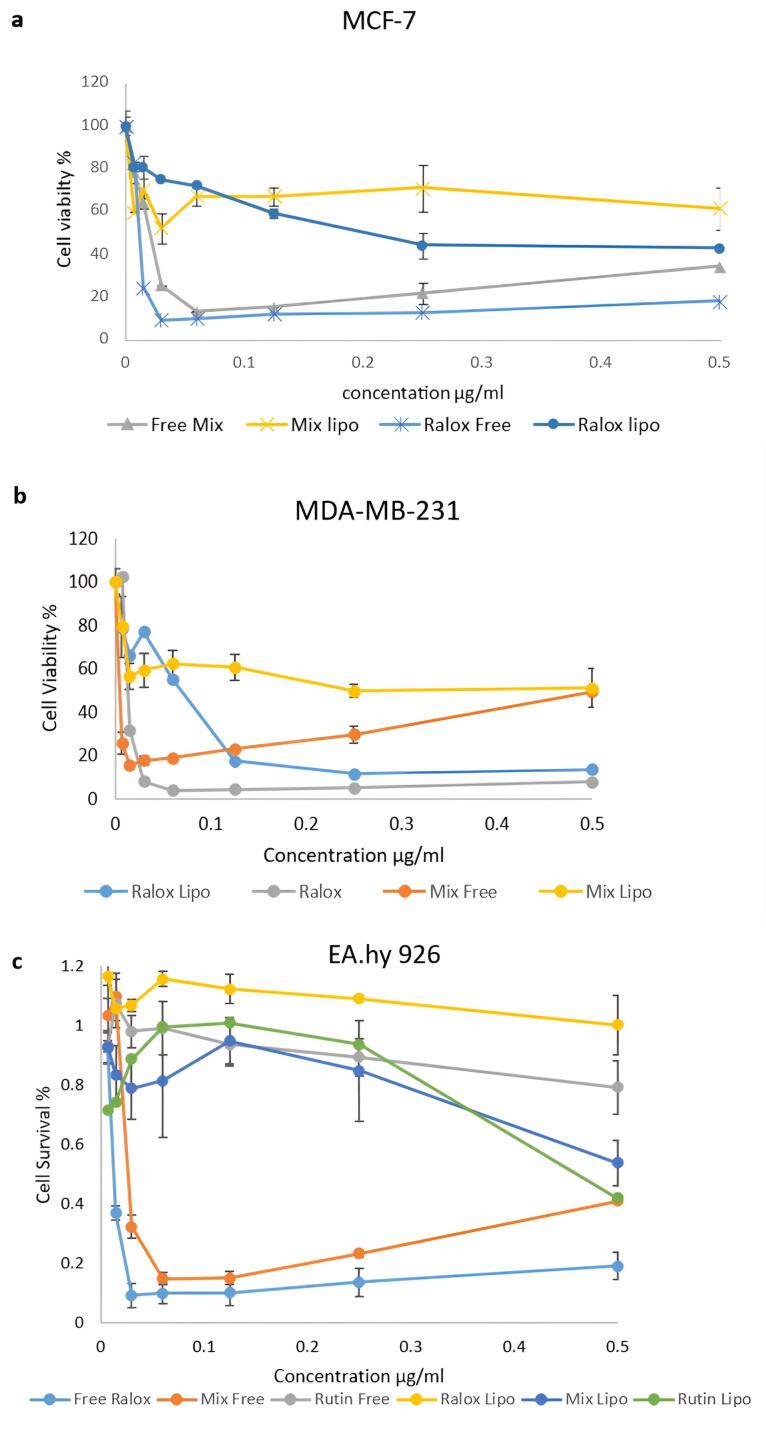
Chemosensitivity curves of (a) MCF-7, (b) MDA-MB-231, and (c) EA. hy926 cells exposed to RLX, liposomal RLX, rutin, Rutin Lipo, free mix or mixed Lipo for 72 hours. All values are averages of triplicates + SDs

 The cytotoxic effects of RLX at various concentrations on MCF-7 cells were evaluated in this study using the MTT assay. The IC_50_ values of free RLX and its liposomal form, which inhibited 50% of MCF-7 cell viability, were determined. Figure 8 illustrates the chemosensitivity of the MTT curves for (a) MCF-7, (b) MDA-MB-231, and (c) EA. hy926 cells following 72 hours of exposure to RLX, liposomal RLX, rutin, rutin Lipo, free mix, or mixed Lipo. Cells cultured in the medium without drug treatment (treated with a vehicle) served as controls.

 The IC_50_ value of free RLX was calculated as 9 µg/mL ± 0.191 after 72 hours. In contrast, the IC_50_ of liposomal RLX was determined to be 40 µg/mL ± 0.13 after the same duration. These results indicate that the IC_50_ of RLX in the nanoliposomal (NL) form was higher than that of the free drug. Additionally, the physical mixture of RLX and rutin exhibited greater cytotoxicity than the liposomal formulation. This suggests that the liposomal preparation mitigated the cytotoxic effects of the RLX-rutin combination.

 Both the incorporation of rutin with RLX in a physical mixture and the encapsulation of RLX within the liposomal formulation reduced the cytotoxicity of RLX against the MCF-7 cell line. For instance, at a concentration of 0.015 µg/mL, the survival rate of MCF-7 cells treated with free RLX alone was 24% ± 0.016, whereas the survival rate for the combination of RLX and rutin as a physical mixture was 64% ± 0.05, and 70% ± 0.09 for liposomal RLX.

####  ER-negative BC cell line (MDA-MB-231)

Figure 8b illustrates the different concentrations of free RLX and its liposomal form used to treat the MDA-MB-231 cell line, along with their corresponding IC_50_ values. After 72 hours of treatment, the concentrations of free RLX and the liposomal form were 6 µg/mL ± 0.14 and 70 µg/mL ± 0.05, respectively. The IC_50_ of RLX in treated cells was 31 ± 0.11 µg/mL. However, upon the addition of rutin, the IC50 increased to 36.0 ± 0.060 µg/mL in the physical mixture and further increased to 55.8 ± 0.008 µg/mL in the liposomal mixture. The addition of rutin did not significantly enhance cytotoxicity against the MDA-MB-231 cell line. Conversely, incorporating RLX into liposomes reduced cytotoxicity by 1.8-fold. These findings indicate that the physical mixture exerted a greater cytotoxic effect on both BC cell lines compared to the liposomal formulation.

####  Normal endothelial cell lines

 To evaluate the selectivity of the NLs, a viability assay was performed on normal cells (Ea. hy926) to assess their potential cytotoxic effects on non-cancerous cells. The MTT assay demonstrated that the NL formulation did not induce significant cytotoxic damage compared to free drugs (Figure 8c). In conclusion, the addition of rutin or the incorporation of RLX within the liposomal formulation reduced cytotoxicity against both BC cell lines while enhancing safety in normal human cells and tissues.

###  In vitro antioxidant assay

**Figure 9:**
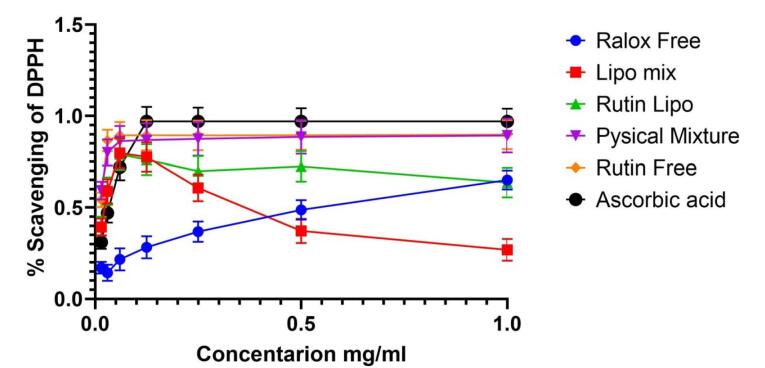
Free radical scavenging activity of RLX, rutin, the mixed solution, and RLX, rutin, and mixed NLs at different concentrations. Ascorbic acid was used as a standard for generating a radical scavenging ability calibration curve

 The free radical scavenging activity of RLX, rutin, their mixed solution, and RLX-, rutin-, and mixed-loaded NLs at various concentrations were evaluated. Ascorbic acid was used as a reference to assess radical scavenging capacity (Figure 9). Free radical scavenging ability is a key indicator of antioxidant activity, which plays a crucial role in reducing oxidative stress and enhancing therapeutic efficacy in drug delivery systems.

 The DPPH radical scavenging assay was conducted, with the results presented in Figure 9. The scavenging activity of DPPH was maximal at 1 mg of RLX/rutin NLs, RLX/rutin solution, RLX, and rutin. As shown in Figure 9, rutin and the physical mixture exhibited enhanced antioxidant effects, whereas RLX alone demonstrated no antioxidant activity. Notably, the mixed-loaded NLs displayed antioxidant activity, indicating a potential enhancement in the combined formulation. The methanolic solution containing RLX and rutin exhibited greater antioxidant activity than the corresponding non-combined solution, suggesting a synergistic effect of co-loading both compounds. The increased antioxidant capacity of mixed-loaded NLs highlights their potential as a multifunctional delivery system, offering both sustained drug release and antioxidant protection. This property could be valuable in mitigating oxidative stress-related damage in various therapeutic applications.

###  Migration results 

**Figure 10:**
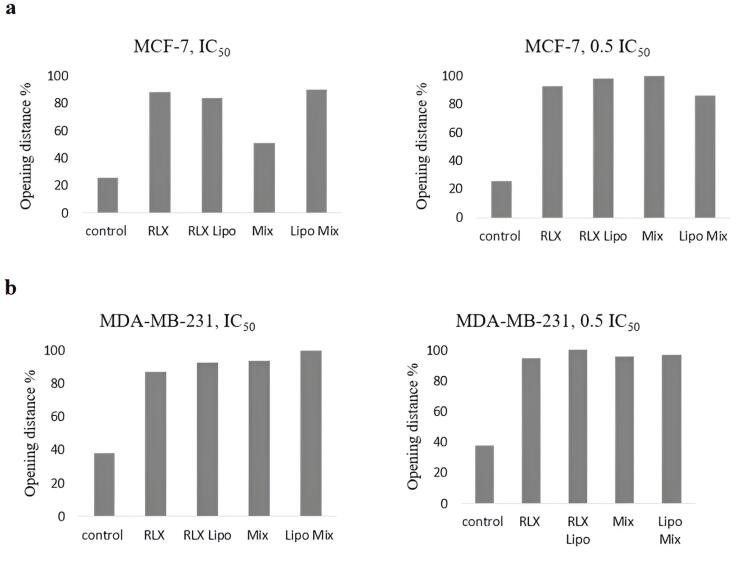
Influence of rutin on the migration and invasion of cells. Representative images of the cell migration assay. (a) Migration of MCF-7 and (b) MDA-MB-231 cells 72 hours after wounding in the different groups. The extent to which all the formulations and free drugs inhibited cell migration was calculated

 The effects of rutin on cell migration and invasion were investigated. Figure 10a depicts the migration of different groups of MCF-7 cells after a 72-hour incubation. Figure 10b shows the migration of MDA-MB-231 cells across the Matrigel surface following a 72-hour scratch assay in various groups. The anti-migration rates of MCF-7 and MDA-MB-231 cells treated with RLX and the mixed-loaded NL formulations at IC_50_ and half-maximal inhibitory concentrations were higher than those of the control group.

**Figure 11:**
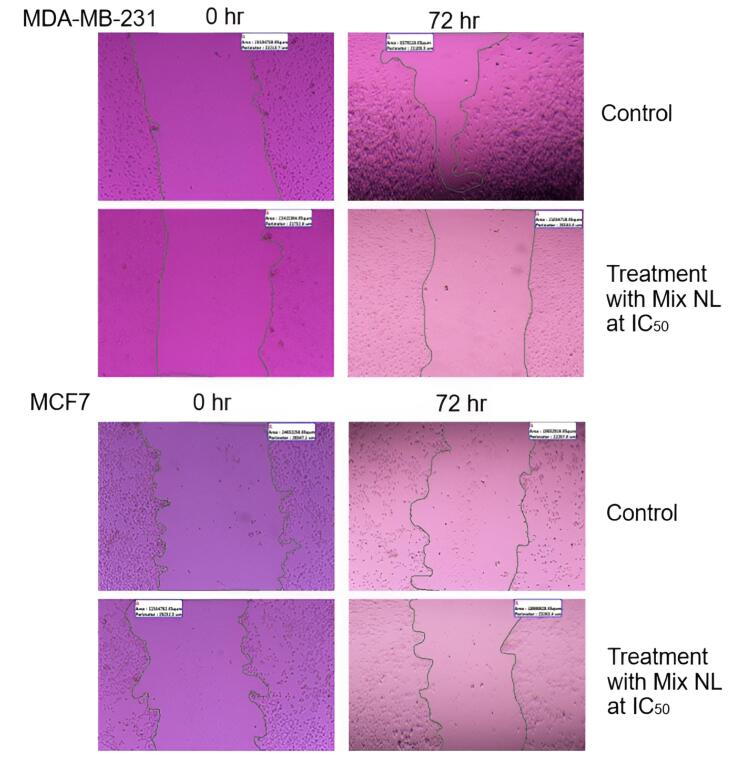
Wound closure area (2 μm) of both BC cell lines treated with mixed NL

 The left image of the MDA-MB-231 cell line (Figure 11) represents the initial condition or an early stage following the wound. The right image, taken later point, demonstrates partial wound closure. Measurements of the wound area and perimeter indicate a progressive decrease over time, illustrating the healing process.

 Similarly, for the MCF7 cell line, the left image depicts the wound at its initial stage, while the right image shows partial closure at a subsequent time point. The provided measurements of area and perimeter further highlight these temporal changes.

 MDA-MB-231 cells, known for their higher aggressiveness and metastatic potential, exhibit different wound-healing behavior compared to the less aggressive MCF7 cells. Although both cell lines show some degree of wound closure, the rate of healing may vary. The concentration at which a therapy inhibits 50% of cell viability is referred to as the IC_50 _concentration. The images suggest that the NL combination affects the wound-healing process in both cell lines, though to varying extents. This assay is commonly used to evaluate cell migration and proliferation.

 By analyzing changes between the initial and later time points, the influence of the therapy on these cellular processes can be assessed. The images indicate that at their respective IC_50_ values, the NL combination impacts wound healing in both MDA-MB-231 and MCF7 cell lines. Since wound closure is closely linked to cell migration and proliferation, its modulation may reflect the therapeutic effect. To draw more definitive conclusions, additional quantitative and statistical analyses are necessary to compare the treatment’s efficacy under controlled conditions.

 The formulation of innovative NLs for the co-delivery of pharmaceuticals is a complex process influenced by various factors. These parameters are crucial in defining nanoparticle properties and drug loading efficiency, ultimately impacting the overall quality of the resulting formulations.^56^

 The findings revealed significant differences in EE% and particle size between formulations using 100% chloroform as the solvent and those using a 75% methanol: chloroform mixture. The increase in EE% from 51.98% to 91.29% for RLX and from 67.84% to 78.12% for rutin aligns with the findings of Ansari et al ^57^ who investigated the effect of solvents on nanoparticle characterization. Additionally, the polarity of the solvents influenced encapsulation efficiency, surface charge, and PDI, affecting the characterization of organic solvent-based formulations. All formulations prepared fell within the optimal limits for size, charge, and PDI.^58,59^ Moreover, the findings demonstrated that co-loading rutin with RLX in NLs improved nanoparticle stability. These results are consistent with previous studies,^60^ which also reported enhanced nanoparticle stability through co-loading.

 Co-loaded NLs carrying RLX exhibited a distinct biphasic release profile, characterized by an initial burst release followed by a second phase with a significantly reduced RLX release rate. This release pattern aligns with findings from previous studies employing similar formulation methods and conditions.^46,61^ TEM analysis confirmed that the NLs were uniformly sized and spherical, consistent with earlier reports using identical preparation methods.^46,60-62^

 The primary rationale for RLX therapy in ER-positive breast tumors lies in its antiestrogenic effect via the ER-dependent pathway, initiated by the formation of the RLX-ER complex, which inhibits estrogen binding to the receptor. Compelling evidence suggests that tamoxifen exhibits multicellular, non-ER-related actions not only in BC but also in other malignancies, such as hepatocellular carcinoma and lung cancer.^63^

 The results of this study provide strong evidence for a non-ER-targeted mechanism, as similar cytotoxic effects were observed in ER-positive (MCF-7), ER-negative (MDA-MB-231), and normal-like cell lines. Numerous studies have demonstrated that RLX influences breast, liver, and prostate cancer cells independently of estrogen receptors. RLX directly binds to the aryl hydrocarbon receptor (AhR), a molecular target that induces apoptosis in both ER-negative mouse and human hepatoma cells, as well as in triple-negative MDA-MB-231 BC cells, while sparing nontrans formed mammary cells.^64^ In vivo xenograft studies indicate that RLX inhibits TNBC growth.^40^ Furthermore, RLX has been shown to exert an alternative mechanism of action in ER-negative cell lines, leading to a 27-fold decrease in EGFR expression and a 70% reduction in Ki67 expression. This process inhibits tumor cell proliferation and promotes apoptosis through caspase-3 activation. Additionally, RLX induces apoptosis in androgen-independent human prostate cancer cell lines.^65^ The literature supports the cytotoxicity studies presented here, reinforcing the investigation of RLX as a potential non-ER-targeted selective estrogen receptor modulator (SERM) in both ER-positive and ER-negative cells. This study provides compelling evidence that a nanoliposome formulation containing RLX reduces cytotoxicity in both cell types, supporting the findings of Oliveira et al^66^ who developed a novel etidocaine formulation that enables sustained release while mitigating cytotoxic effects.

 MTT cell viability assays were used to assess the cytotoxic effects of RLX and rutin when administered as free drugs, physical mixtures, or NLs, using the normal endothelial cell line EA. hy926. The results suggest a favorable safety profile for the nanoliposomal formulations compared with free drugs. Specifically, ‘Ralox Lipo’ and ‘Rutin Lipo’ exhibited greater cell viability at increasing concentrations, indicating reduced toxicity. In contrast, ‘Free Ralox’ and ‘Rutin Free’ led to significant decreases in cell viability with increasing concentrations, suggesting heightened toxicity. The ‘Mix Lipo’ group also maintained greater cell viability at all tested concentrations, underscoring the protective effect of liposomal encapsulation. Ideally, for normal cell lines, maintaining high cell viability even at elevated drug concentrations is desirable, a goal achieved by liposomal formulations. This finding highlights the enhanced safety of liposomal carriers, as they are designed to specifically target cancer cells while minimizing damage to normal cells. The liposomal formulations of RLX and rutin, both individually and in combination, demonstrate potential for safer therapeutic applications by preserving healthy cell integrity during cancer treatment.

 The effects of different RLX and rutin formulations on the migration of MCF-7 and MDA-MB-231 BC cells were evaluated using a migration assay. The results indicate that these formulations significantly influence cell motility, an important factor in metastatic potential. As expected, the control groups exhibited the least migration inhibition. In comparison, the RLX-loaded liposomal formulation (RLX Lipo) and the combined liposomal mixture (Lipo Mix) demonstrated greater inhibitory effects on cell migration at both the IC₅₀ and 0.5 IC₅₀ concentrations, suggesting their potential in reducing cancer cell metastasis. Free RLX and the physical mixture (Mix) displayed intermediate effects, while the liposomal formulations showed a pronounced improvement in migration inhibition. These findings suggest that liposomal encapsulation of RLX and rutin not only enhances solubility and safety, as previously discussed, but also enhances their therapeutic efficacy in preventing cancer cell migration, an essential factor in controlling BC metastasis.

 Regarding the radical scavenging activity of RLX and rutin formulations, the data indicate that free rutin exhibits superior efficacy, maintaining high inhibition percentages across all concentrations, consistent with its well-documented antioxidant properties. However, liposomal encapsulation of rutin and RLX resulted in a decline in scavenging activity, with a marked decrease at higher concentrations. This effect may be attributed to the encapsulation altering the compounds’ interactions with free radicals. Interestingly, the liposomal mixture of RLX and rutin did not demonstrate the anticipated synergistic effect, instead showing a peak at an intermediate concentration followed by a decline. The physical mixture exhibited the least efficacy, suggesting that the free forms of RLX and rutin might interact more effectively with free radicals than their physically combined counterparts. These results indicate that while liposomal delivery improves targeting and solubility, it may not be the optimal strategy for enhancing the antioxidant activity of RLX and rutin. This underscores the importance of tailoring formulation strategies to meet specific therapeutic objectives.

## Conclusion

 This study successfully developed and characterized PEGylated NLs co-loaded with RLX and rutin, offering a promising drug delivery system for BC treatment. The encapsulation efficiencies of RLX and rutin were 91.28% and 78.12%, respectively, demonstrating effective drug loading. Stability studies confirmed that the NLs maintained their structural integrity for up to two months at room temperature and one month at 4°C. In vitro release the profiles exhibited a biphasic release pattern, with sustained RLX and rutin release over extended periods, suggesting the potential for reduced dosing frequency and minimized toxicity.

 Cytotoxicity assays against MCF-7 and MDA-MB-231 BC cell lines revealed that the liposomal formulations reduced toxicity compared to free drugs while retaining significant anticancer activity. Additionally, the RLX-rutin NLs enhanced antioxidant activity and inhibited cancer cell migration, highlighting their potential role in preventing metastasis. The improved safety observed in standard cell lines suggests selective therapeutic action. Transmission electron microscopy confirmed the uniform spherical morphology of the NLs, aligning with optimal nanoparticle design for biomedical applications.

 This study underscores the potential of nanoliposomal co-delivery systems to enhance the therapeutic index of conventional drugs and natural antioxidants. Future research should focus on evaluating vivo efficacy and pharmacokinetics to validate clinical applicability. These findings contribute to the advancement of nanotechnology-based strategies for targeted, sustainable, and safer BC therapies.

## Competing Interests

 The authors declare that they have no conflict of interest.

## Ethical Approval

 Not applicable.
